# Risk factors associated with adverse cardiovascular events during orthotopic liver transplantation and the early post-transplant period

**DOI:** 10.3325/cmj.2025.66.194

**Published:** 2025-06

**Authors:** Nataša Višković, Filipčić, Katarina Tomulić Brusich, Milan Milošević, Sonja Perkov, Miroslav Župčić, Tajana Filipec, Kanižaj

**Affiliations:** 1 Department of Cardiology and Surgery, Special Hospital Agram, Zagreb, Croatia; 2 Department of Anesthesiology, Intensive Care and Pain Medicine, University Hospital Centre Rijeka, Rijeka, Croatia; 3 Department of Environmental Health, Occupational and Sports Medicine, Andrija Štampar School of Public Health, Zagreb, Croatia; 4 Department for Medical Biochemistry and Laboratory Medicine, University Hospital Merkur, Zagreb, Croatia; 5 Clinic of Anesthesiology, Reanimatology and Intensive Care Medicine, Clinical Hospital Centre Rijeka, Rijeka, Croatia; 6 Department of Clinical Sciences II, Faculty of Health Studies, University of Rijeka, Rijeka, Croatia; 7 Department of Gastroenterology, University Hospital Merkur, School of Medicine, University of Zagreb, Zagreb, Croatia

## Abstract

**Aim:**

To evaluate the incidence of severe adverse cardiovascular events during orthotopic liver transplantation and the early post-transplant period, and to determine the factors associated with these events.

**Methods:**

We retrospectively reviewed the medical records of 291 patients who underwent orthotopic liver transplantation at University Center Merkur, Zagreb, Croatia, from 2013 to 2015. Data on corresponding donor characteristics were sourced from the Eurotransplant database. Severe adverse cardiovascular events were defined as myocardial infarction, malignant arrhythmia, pulmonary embolism, cardiac failure, cardiac arrest, and death.

**Results:**

The incidence of severe adverse events was 8.9%. Univariate analysis demonstrated a positive predictive value linking severe adverse cardiovascular events with metabolic liver disease, an elevated model for end-stage liver disease, an international normalized ratio, and the preoperative administration of vasopressors. Multivariate analysis identified significant predictors of adverse events: liver metabolic diseases (odds ratio [OR] 4.88; 95% confidence interval [CI] 1.48-16.09; *P* = 0.009), donor arterial hypertension (OR 2.68; 95% CI 1.01-7.18; *P* = 0.049), prolonged cold ischemia duration (OR 1.22; 95% CI 1.01-1.49; *P* = 0.044), and increased recipient age (OR 1.09; 95% CI 1.03-1.16; *P* = 0.005).

**Conclusion:**

The occurrence of severe adverse cardiovascular events is markedly greater than previously documented. We propose the use of a scoring system including known risk variables to identify patients at risk of adverse cardiovascular events, hence improving surveillance for an early intervention.

Registration number: ISRCTN16660149

Liver transplantation is the final curative procedure for end-stage liver disease (ESLD). Many chronic liver diseases, with cirrhosis as the leading cause, can lead to ESLD (1). Liver disease considered for orthotopic liver transplantation (OLT) can be divided into six categories: alcoholic liver cirrhosis, primary liver tumors (hepatocellular and cholangiocellular carcinoma), cirrhosis caused by viruses (hepatitis B and C), cholestatic liver disease (primary or secondary biliary cirrhosis, primary sclerosing cholangitis), metabolic liver disease (nonalcoholic steatohepatitis [nonalcoholic fatty liver disease, NAFLD], hemochromatosis, Wilson’s disease), and other liver diseases (autoimmune hepatitis, polycystic liver disease, neuroendocrine tumor metastases, etc). Most liver diseases, except for primary liver tumors, are preventable and curable. These major groups commonly overlap, since chronic liver disease caused by alcohol or a virus can promote hepatocarcinogenesis (2-13). Metabolic liver diseases (ie, NAFLD) bring the largest burden of cardiovascular disease into OLT, sharing the same pathophysiological mechanisms with cryptogenic cirrhosis (14).

Since 2002, the scoring system for patients on the liver transplant list has been the Model for End-Stage Liver Disease (MELD). It estimates 3-month mortality for patients with chronic liver disease (15). Its greatest value lies in the identification of patients that may benefit the most from transplant procedures (15,16).

Cadaveric donors still represent most donors worldwide and can be divided into brain-dead and cardiac-dead donors (17). Since the demand for donors outgrows the eligible pool of donors, the concept of extended-criteria donors (ECD) has become popular. Compared with standard criteria donors, ECD bring an increased burden of complications into the transplant procedure, whether it is a greater risk of disease transfer or technical complications (18,19). The criteria for an ECD for the Eurotransplant region have been defined, and each recipient must be carefully paired with an ECD (20,21). The burden that donors bring to liver transplantation is defined through the donor risk index (DRI), which estimates the recipient's 3-month survival after OLT (22,23). While there is no clear cutoff value for DRI that defines an ideal organ, 1.7 is thought to be the line between high-quality and low-quality grafts (23). Matching high-MELD recipients with poor DRI donors does not increase the relative risk of organ dysfunction, and this type of pairing is generally accepted (24).

Liver transplantation is one of the most complex procedures in hepatobiliary surgery, carrying a high risk for cardiac death and complications within 30 days postoperatively. A significant number of patients with ESLD have cardiovascular comorbidities, which may affect morbidity and mortality following OLT. Cardiac assessment of liver transplant recipients should adhere to the 2014 American College of Cardiology and the American Heart Association guidelines regarding perioperative cardiovascular evaluation and care for patients having noncardiac surgery (25). The purpose of cardiac examination for recipients is to stratify risk and identify asymptomatic cases of heart illness (26). Nevertheless, even patients without cardiac symptomatology can suffer from adverse cardiac events during this complex procedure. Adverse cardiac events during OLT and post-procedure are classified as mild or severe. Mild adverse events encompass arrhythmias that resolve spontaneously or with pharmacological intervention, excluding severe ventricular rhythm disturbances, and include non-cardiac hypotension that persists beyond 24 hours without response to vasopressor therapy. Severe adverse cardiac events include malignant/ventricular arrhythmia, cardiac decompensation, myocardial infarction, pulmonary embolism, and mortality.

Only several studies have examined the occurrence of adverse cardiac events in liver transplant recipients. Research investigating the relationship between donor and recipient characteristics regarding these events is limited (27-29). Identifying the characteristics associated with donors and recipients that contribute to adverse cardiac events during OLT and the early post-transplant period may promote more prudent donor-recipient matching. This may reduce morbidity and mortality, enhance graft use and longevity, diminish complications, and lessen overall treatment costs.

This study aims to determine the overall incidence of adverse cardiovascular events in patients undergoing liver transplantation and in the early post-transplant period. Moreover, it aims to identify the factors related to donors and recipients that contribute to the occurrence of severe adverse cardiovascular events. The 2014 ESC/ESA Guideline for Perioperative Cardiovascular Management for Noncardiac Surgery indicates that the risk of cardiovascular events during major abdominal procedures, including liver transplantation can reach 5% (25). Consequently, we hypothesized that the incidence of serious cardiovascular events in our study would be below 5%.

## Patients and methods

We retrospectively reviewed the data on patients undergoing liver transplantation at University Hospital Merkur, Zagreb, Croatia, from January 1, 2013 to December 31, 2015. The study was approved by the Ethics Committee of University Hospital Merkur. The exclusion criteria were age lower than 18 years, liver retransplantation, acute liver failure, living-donor transplantation, and multivisceral transplantation.

Recipient data were collected from medical patients’ records during the pre-transplant evaluation and hospitalization for the OLT procedure. Data were collected on age, sex, blood type, liver disease as the indication for liver transplantation, comorbidities (cardiovascular disease, renal disease, arterial hypertension, diabetes, hyperlipidemia), smoking status, pre-transplant vasopressor administration, and pre-transplant initiation of renal replacement therapy. MELD was calculated on the day of hospital admission for OLT (laboratory-based or with the exception criteria). MELD mathematically combines three parameters: bilirubin, creatinine, and international normalized ratio (INR), stratifying the patients on the waiting list into the following risk groups: low (<15), intermediate (15-25), and high (>25), with a maximum value of 40.

Donor characteristics were obtained from the Eurotransplant online database encompassing organ donor reports detailing age, height, body mass index, the cause of death, donor location, donation of a whole organ or split-liver donation, race, cold ischemia time, anti-HBc status, donor comorbidities, cardiopulmonary resuscitation events, and hypotensive episodes. Additionally, the DRI was calculated using the following factors: age, height, race, cause of death (cerebrovascular accident, trauma, anoxia, and others), donation after cardiac death, split transplantation, cold ischemia time, and the donor's location in relation to the transplant center (local, regional, or national) (22,23) to quantitatively predict the risk of post-transplantation graft failure in OLT.

We also recorded intraoperative use of vasopressors during OLT and within the first 24 hours, the duration of mechanical ventilation, ICU length of stay, and the occurrence of adverse cardiovascular events within 30 days post-OLT. Adverse cardiovascular events were categorized into two groups: mild and severe, based on established criteria. Mild adverse cardiovascular events included hypotension of non-cardiac origin and arrhythmogenic events (non-ventricular) that resolve spontaneously or following fluid correction or medical intervention. Severe adverse cardiovascular events consisted of ventricular rhythm disorders, acute myocardial infarction, heart failure, pulmonary embolism, cardiac arrest, and death.

Patients were prepared for OLT according to the institutional protocol of University Hospital Merkur. Following the routine preoperative assessment for OLT, general anesthesia was administered by two experienced anesthesiologists. Standard hemodynamic monitoring, including arterial and central venous lines, was conducted. However, it was augmented if required by arterial pulse contour analysis and pulmonary arterial pressure monitoring. Volume status, electrolytes, anemia, thrombocytopenia, and coagulation abnormalities were corrected according to laboratory results and clinical findings. Post-transplant, patients who remained intubated and on mechanical ventilation were transferred to the intensive care unit (ICU). The ICU specialist performed weaning and extubation. The duration of ICU stay was contingent upon the patient's condition, and after they met the criteria, they were discharged to the surgical ward. All relevant data were gathered by a physician overseeing the study. Data were gathered in written form and converted to digital form. Data confidentiality was guaranteed in accordance with ethical standards, declarations, and local legislation.

### Statistical methods

The incidence of individual adverse outcomes is expressed as absolute frequencies and 95% confidence intervals (95% CI). Categorical variables are presented as absolute frequencies and relative frequencies, while continuous values are presented as medians and interquartile ranges (IQR). The correlation of individual recipient and donor characteristics with the development of adverse cardiovascular events in liver transplant patients within 30 days was evaluated using Kendall's tau-b correlation coefficient. All values that were significant at the *P* < 0.100 level were entered as predictors in a multivariate regression model (binary logistic regression, in which the dependent variable was 1 = adverse event within 30 days and 0 = no adverse event). *P* values less than 0.05 were considered significant. The analysis was conducted with MedCalc® Statistical Software, version 20.112 (MedCalc Software Ltd, Ostend, Belgium).

## Results

The characteristics of the recipients are presented in Table 1. The most common diagnosis as an indication for liver transplantation was alcoholic cirrhosis (118 or 40.5%). Most recipients were 40-65 years old (75.9%), with the median age of 58.0 (IQR 52.0-63.0) years, and predominantly male (72.5%). Diabetes was the most prevalent comorbidity, occurring in 26.8% of the recipients. The laboratory MELD reported to Eurotransplant was 15.0 (IQR 12.0-19.0).

**Table 1:** The characteristics of liver transplant recipients*

Characteristic	
**Blood group type: n (%)**	
A	117 (40.2)
AB	21 (7.2)
B	54 (18.6)
O	99 (34.0)
Rh positive: n (%)	228 (78.4)
Liver disease type: n (%)	
alcoholic disease	118 (40.5)
primary tumors	20 (6.9)
viral cirrhosis	23 (7.9)
overlapping	74 (25.4)
cholestatic diseases	23 (7.9)
metabolic and other diseases	33 (11.3)
Male sex: n (%)	211 (72.5)
Age (years): median (IQR)	58.0 (52.0-63.0)
BMI (kg/m^2^): median (IQR)	26.3 (23.7-29.4)
Creatinine: median (IQR)	75.0 (59.0-104.0)
Bilirubin: median (IQR)	41.0 (22.0-82.0)
INR: median (IQR)	1.5 (1.3-1.6)
Heart diseases: n (%)	41 (14.1)
Hypertension: n (%)	71 (24.4)
Diabetes mellitus: n (%)	78 (26.8)
Smoking: n (%)	52 (17.9)
Hyperlipoproteinemia: n (%)	9 (3.10)
Preoperative vasopressors: n (%)	2 (0.7)
Preoperative hemodialysis: n (%)	2 (0.7)
MELD on transplantation day: median (IQR)	15.0 (12.0-19.0)
MELD reported to Eurotransplant: median (IQR)	15.0 (12.0-19.0)
MELD real with exclusion criteria reported to Eurotransplant: median (IQR)	18.0 (13.0-22.0)

*Abbreviations: IQR – interquartile range; BMI – body mass index; INR – international normalized ratio: MELD – Model for End Stage Liver Disease

The median donor age was 59.0 (47.0-69.0) years, with arterial hypertension as the most common comorbidity (132 or 45.4%). The median DRI was 1.63 (1.38-1.91), and the median duration of cold ischemia was 6.83 hours (5.02-8.68) (Table 2). In 64.9% of patients, donors completely matched the recipients’ blood type. The ICU length of stay was 4.0 (3.0-6.0) days, and overall hospital survival was reported in 274 (94.2%) patients.

**Table 2:** Donor and intensive care unit (ICU) procedure characteristics*

Characteristic	
Rh positive: n (%)	238 (81.8)
Male sex: n (%)	186 (63.9)
Cause of death: n (%)	
anoxia	6 (2.1)
cerebrovascular accidents	216 (74.2)
other	15 (5.2)
trauma	54 (18.6)
Positive anti HBc: n (%)	28 (9.6)
DRI groups >1.7: n (%)	125 (43.0)
Heart diseases: n (%)	45 (15.5)
Hypertension: n (%)	132 (45.4)
Diabetes mellitus: n (%)	29 (10.0)
Smoking: n (%)	47 (16.2)
Same blood type between donor/recipient: n (%)	189 (64.9)
Intraoperative vasopressors: n (%)	163 (56.0)
Postoperative vasopressors 24h: n (%)	95 (32.6)
Hemodialysis intra/postoperative: n (%)	14 (4.8)
Hospital survival: n (%)	274 (94.2)
Age (years): median (IQR)	59.0 (47.0-69.0)
BMI (kg/m^2^): median (IQR)	26.0 (24.0-28.0)
CIT (hours): median (IQR)	6.83 (5.02-8.68)
DRI: median (IQR)	1.63 (1.38-1.91)
Highest potassium levels (mmol/L): median (IQR)	4.90 (4.30-5.70)
Length of stay in ICU (days): median (IQR)	4.00 (3.00-6.00)
Mechanical ventilation (hours): median (IQR)	10.0 (6.00-18.0)

*Abbreviations: IQR – interquartile range; BMI – Body mass index; Rh – rhesus factor; HBc – hepatitis B core antigen; CIT – cold ischemia time; DRI – donor risk index; ICU – intensive care unit.

The incidence of serious adverse events at 30 days was 8.9% (95% CI 6.1%-12.6%) (Table 3). At the univariate level, metabolic and other liver diseases, higher INR, higher laboratory MELD reported to Eurotransplant, and the use of preoperative vasopressors were significantly positively correlated with the number of adverse events (Table 4). The number of adverse cardiovascular events in liver transplant recipients was significantly positively correlated with vasopressor administration in the donor in the first 24 hours postoperatively (Table 5). All predictor variables that had a univariate significance level of less than 0.1 were included in the multivariate regression model to predict adverse cardiovascular events (Table 6). The regression model was statistically significant at the *P* = 0.001 level, correctly classifying 91.1% of participants (R^2^ = 23.3%). In the multivariate setting after controlling for the influence of all other variables in the regression model, significant predictors of adverse events were liver metabolic and other diseases (odds ratio [OR] 4.88; 95% confidence interval [CI] 1.48-16.09; *P* = 0.009), donor arterial hypertension (OR 2.68; 95% CI 1.01-7.18, *P* = 0.049), longer duration of ischemia (OR 1.22; 95% CI 1.01-1.49, *P* = 0.044), and older recipient age (OR 1.09; 95% CI 1.03-1.16, *P* = 0.005).

**Table 3:** The incidence of adverse cardiovascular events in liver transplant recipients

Adverse events		N	%	95% confidence interval	
Intraoperative	without	121	41.6	36.0	47.3
	mild	163	56.0	50.3	61.6
	severe	7	2.4	1.1	4.7
Early postoperative (in 30 days)	without	173	59.5	53.7	65.0
	mild	99	34.0	28.8	39.6
	severe	19	6.5	4.1	9.8
Total (+30 days)	without	107	36.8	31.4	42.4
	mild	158	54.3	48.6	60.0
	severe	26	8.9	6.1	12.6

**Table 4:** Correlation of the total number of adverse cardiovascular events in liver transplant recipients with their individual characteristics during postoperative 30 days*

Characteristic		Adverse events in 30 days
Alcoholic disease	correlation coefficient	-0.038
	P	0.519
Primary tumors	correlation coefficient	0.010
	P	0.863
Viral cirrhosis	correlation coefficient	-0.092
	P	0.118
Overlapping	correlation coefficient	0.011
	P	0.855
Cholestatic diseases	correlation coefficient	-0.002
	P	0.967
Metabolic and other diseases	correlation coefficient	**0.116**
	P	**0.048**
Age (years)	correlation coefficient	0.139
	P	0.004
Female sex	Correlation coefficient	0.023
	P	0.695
BMI	correlation coefficient	-0.001
	P	0.990
Creatinine	correlation coefficient	0.080
	P	0.099
Bilirubin	correlation coefficient	0.050
	P	0.296
INR	correlation coefficient	**0.107**
	P	**0.034**
MELD on transplantation day	correlation coefficient	0.083
	P	0.093
MELD reported to Eurotransplant	correlation coefficient	**0.115**
	P	**0.019**
MELD real with exclusion criteria reported to Eurotransplant	correlation coefficient	0.085
	P	0.086
Heart diseases	correlation coefficient	0.087
	P	0.139
Hypertension	correlation coefficient	-0.010
	P	0.870
Diabetic mellitus	correlation coefficient	-0.054
	P	0.362
Smoking	correlation coefficient	-0.052
	P	0.378
Preoperative vasopressors	correlation coefficient	**0.120**
	P	**0.041**
Preoperative hemodialysis	correlation coefficient	-0.026
	P	0.657

*Abbreviations: BMI – body mass index; INR – international normalized ratio; MELD – Model for End Stage Liver Disease.

**Table 5:** Correlation of the total number of adverse cardiovascular events in liver transplant recipients with individual characteristics of the donor during 30 postoperative days*

Characteristic		Adverse events in 30 days
Age (years)	correlation coefficient	-0.019
	P	0.702
Female sex	correlation coefficient	0.016
	P	0.792
BMI	correlation coefficient	-0.019
	P	0.702
Cold ischemia time (hours)	correlation coefficient	0.079
	P	0.066
Anti HBc	correlation coefficient	-0.061
	P	0.296
DRI	correlation coefficient	0.005
	P	0.914
Heart diseases	correlation coefficient	0.033
	P	0.578
Hypertension	Correlation coefficient	0.102
	P	0.083
Diabetic mellitus	correlation coefficient	-0.024
	P	0.686
Smoking	correlation coefficient	0.092
	P	0.118
Same blood type with receiving patient	Correlation coefficient	-0.048
	P	0.417
Intraoperative vasopressors	correlation coefficient	0.108
	P	0.067
Highest potassium levels	Correlation coefficient	-0.035
	P	0.471
Postoperative vasopressors	correlation coefficient	**0.142**
	P	**0.016**

*Abbreviations: BMI – body mass index; anti HBc – anti hepatitis B core antigen; DRI – Donor Risk Index.

**Table 6:** Multivariate regression model of prediction of adverse cardiovascular events in liver transplant patients: binary logistic regression

	Odds ratio	95% confidence interval		P
		upper	lower	
Liver disease type: metabolic and other	**4.88**	**1.48**	**16.09**	**0.009**
Patients age (years)	**1.09**	**1.03**	**1.16**	**0.005**
Creatinine level	1.00	0.99	1.00	0.590
Patients INR	1.07	0.40	2.90	0.889
MELD reported to Eurotransplant	1.07	0.99	1.16	0.090
Cold ischemia time (donor)	**1.22**	**1.01**	**1.49**	**0.044**
Preoperative vasopressors (patients)	5.28	0.18	151.54	0.331
Hypertension (donor)	**2.68**	**1.01**	**7.18**	**0.049**
Intraoperative vasopressors (donor)	1.11	0.36	3.38	0.856
Postoperative vasopressors (donor)	1.74	0.63	4.84	0.286

*Abbreviations: INR – international normalized ratio; MELD – Model for End Stage Liver Disease.

## Discussion

The incidence of serious adverse cardiovascular events in this study was 8.9%, markedly above the anticipated 5% as per the 2014 ESC/ESA Guidelines for Perioperative Cardiovascular Management for Noncardiac Surgery. However, it has to be noted that these guidelines primarily assess the risk of fresh myocardial infarction and/or cardiac death within 30 days after surgery, not considering the patient's preoperative condition, comorbidities, urgency, and the extent of the procedure. Nonetheless, the recommendations were not designed for patients with organ failure or multiple organ system failures, and it was presumed that the surgical procedure would occur promptly or quickly after the preoperative assessment. As is widely acknowledged, the waiting period for OLT recipients may extend from several months to over a year (30,31).

The definition of adverse cardiovascular events in the guidelines also differs greatly from our definition. In addition to myocardial infarction and cardiac mortality, we also incorporated ventricular rhythm abnormalities, cardiac failure, and pulmonary embolism, as our objective was to discover the maximum number of significant adverse events. A larger patient sample is necessary for a meaningful evaluation, as these two occurrences have a considerably lower incidence relative to all adverse events.

During the reperfusion stage of OLT, rhythm disorders are prevalent, particularly bradycardia and asystole, necessitating pharmacological intervention and perhaps brief cardiac resuscitation. If timely and correctly treated, these events are transient and generally do not result in any lasting effects. However, when analyzing surgical complications, Dindo et al classified these events as significant with potentially poor outcomes (32). Consequently, we incorporated these events into our study, which undoubtedly elevated our overall incidence of unfavorable cardiovascular events.

The incidence of adverse cardiovascular events linked to liver transplantation, especially serious events, is not widely investigated, and most studies use different definitions of these events, different observation periods, and different populations. Konerman et al (33) systematically reviewed 29 studies with a total of 57 493 recipients but used very inconsistent criteria for adverse cardiovascular events. The incidence of different adverse cardiovascular events in 6 months was 1%-41% (33). The 30-day incidence of serious adverse cardiovascular events in the study by Van Wagner et al was 8%, which aligns with our findings. However, contrary to our study, they included atrial fibrillation in the serious events group (34).

These studies, conducted in the USA, differ from ours in certain basic recipient characteristics. The difference in liver disease prevalence due to population characteristics, as well as the organization and availability of health care, differ in Croatia’s public health system compared with the US’s mostly private and insurance-based health care. Additionally, the location of the donor significantly affects the duration of cold ischemia, influencing DRI and the incidence of adverse events.

The intraoperative incidence of significant adverse events in our study was 2.4%, increasing to 6.5% within the first 30 days post-OLT. This increase was associated with a significant therapeutic intervention, the emergence of *de novo* metabolic syndrome, and the deterioration of the current condition. Immunosuppressive therapy should not be held accountable, as the onset of metabolic problems related to it is not often recognized until six months after OLT (35).

In our study population, the traditional cardiovascular risk factors were not significantly present, which indicates a low anticipated risk of coronary disease and cardiovascular events. The prevalence of metabolic and other disorders, mostly represented by NAFLD and cryptogenic cirrhosis – two conditions predominantly associated with metabolic syndrome – was just 11.3%. In contrast, the incidence of metabolic syndrome worldwide has become significantly associated with liver disease indicated for OLT over the past 30 years, with a prevalence ranging from 10% to 40% (36). Our population's lower prevalence of metabolic syndrome may be explained by the Mediterranean diet and effective diabetes management. However, recent lifestyle and nutritional changes, along with rising obesity rates, suggest that in the coming years the Croatian population may align more closely with global trends regarding this syndrome (36).

In our study, the predominant reason for OLT was alcohol-induced ESLD. This group of patients may develop alcohol cardiomyopathy, alternatively referred to as cirrhotic cardiomyopathy. It is a possibly reversible and typically asymptomatic condition. During significant cardiovascular stress, such as transplant procedures, it may present as a hyperdynamic condition, characterized by impaired diastolic relaxation, reduced cardiac contractility, and electrophysiological abnormalities, typically QT prolongation (37). The prevalence of coronary disease in our cohort (14.1%) considerably differs from the published literature. Luca et al report the incidence of 27%. However, recent data estimate this rate to be as high as 32.5% (38). An explanation for such a low rate in our study may be that cirrhotic cardiomyopathy is underestimated as a diagnosis in our center. Cardiac evaluation of recipients is usually done without invasive cardiac testing, unless indicated. Noninvasive assessments, such as the dobutamine stress test, are not conducted because of their relatively strong negative predictive value (31). Numerous studies demonstrate that coronary artery disease is not inherently associated with an elevated risk of adverse cardiac events, and if the condition is well-managed, it should not preclude the transplant process (39,40).

Univariate analysis comparing donor and recipient characteristics identified metabolic liver disease, elevated INR values indicative of liver disease severity, increased laboratory MELD scores, preoperative vasopressor therapy, donor hypertension, and postoperative vasopressor use within 24 hours as significant factors contributing to severe adverse cardiovascular events. The group of metabolic liver diseases (correlation coefficient of 0.116 and *P* = 0.048) showed about 5 times greater incidence of serious adverse events than other types of liver disease. This heterogeneous group includes a wide range of liver diseases with different pathophysiological mechanisms (accumulation disease, NAFLD, and cryptogenic cirrhosis), with an incidence of 11.3%. Our sample was too small to distinguish the contribution of each disease *per se*. Nonetheless, it is certain that other disease types do not demonstrate similar influence and should not be largely regarded as responsible for these unfavorable consequences. From the multivariate model, several properties arose as predictor variables: metabolic liver disease, donor hypertension, the length of cold ischemia time, and older age of recipients. The older age of recipients and their diagnosis of NAFLD as a representative of metabolic disease provide a solid connection between our study and other major studies (34,41).

We reported an incidence of 5.9% for early or in-hospital mortality. This finding aligns with European studies showing in-hospital death rates of up to 6.3% (42,43). Despite older recipient age being a factor, they identify renal impairment as the primary predictive element (44), which is not corroborated by our data. The mortality figures are quite similar despite inconsistent criteria and timeframes, with sepsis identified as the primary cause of mortality, rather than adverse cardiovascular events. However, we believe that predicting and mitigating risk factors for severe cardiovascular events could also reduce early mortality. A scoring system incorporating risk indicators should be devised from the acquired data to identify patients at risk of adverse cardiovascular events, hence facilitating enhanced surveillance for early detection and intervention.

A limitation of the study is the fact that it involved a population transplanted almost a decade ago. Metabolic and other liver diseases, particularly NAFLD, have escalated during this timeframe owing to the anticipated increase in the prevalence of metabolic disorders in our community. It would be intriguing to investigate whether these unfavorable occurrences associated with metabolic liver disease have altered. Nonetheless, our study presents significant findings about adverse cardiovascular events occurring during and following OLT. Given that most studies focus on the US population, our research provides useful insights into the Eurotransplant zone population.

In conclusion, the incidence of serious adverse cardiovascular events is considerable, as it increases morbidity and mortality among liver transplant patients, resulting in prolonged hospitalization and elevated costs. The comparability to published studies is primarily constrained by inconsistent definitions for adverse cardiac events and variations in the studied populations. Given the data obtained, a straightforward scoring system that integrates risk factors should be created, as there are few sufficient and validated scales on this subject.
